# Editorial: Nutritional Strategies to Promote Muscle Mass and Function Across the Health Span

**DOI:** 10.3389/fnut.2020.569270

**Published:** 2020-10-02

**Authors:** Daniel R. Moore, Andrew Philp

**Affiliations:** ^1^Faculty of Kinesiology and Physical Education, University of Toronto, Toronto, ON, Canada; ^2^Health Ageing Division, Garvan Institute of Medical Research, Darlinghurst, NSW, Australia; ^3^St Vincent's Medical School, University of New South Wales Medicine, University of New South Wales Sydney, Sydney, NSW, Australia

**Keywords:** muscle, exercise, metabolism, function, nutrition, performance

Skeletal muscle is a highly plastic tissue, able to remodel in response to its physical demands. This includes growth (i.e., hypertrophy) in response to the application of external forces (e.g., exercise) and loss (i.e., atrophy) in response to the withdrawal of these forces (e.g., detraining, immobilization). Given its central role in converting chemical energy to mechanical work, skeletal muscle is unquestionably important for individuals wishing to excel in athletic competition, effectively navigate rehabilitation settings (e.g., return to play, remobilization after injury), and perform activities of daily living (e.g., maintain functional independence with age). However, this tissue is a major contributor to the basal metabolic rate and is the preferred storage depot for dietary sugars and fats, which positions it as a vital tissue for the maintenance of metabolic health. Thus, maintaining an adequate quantity and quality of skeletal muscle is important for optimal health and performance throughout the lifespan.

This recent special issue on “Nutritional strategies to promote muscle mass and function across the health span” represents a collection of 21 articles, including 12 original research articles, from 130 of the world leaders in the fields of muscle physiology, nutrition, and exercise physiology. A common theme throughout the special issue is the interactive effects of muscle contraction and dietary nutrients, in which exercise can “make nutrition better” and nutrition can improve muscle mass and function. For example, Oikawa et al. highlighted the importance of maintaining muscle activity to help stave off the deleterious effects of “anabolic resistance,” which is the impaired ability to utilize dietary amino acids to support muscle protein synthesis and tissue remodeling that ultimately leads to decrements in muscle mass and function. Importantly, the authors highlight that reduced daily step counts, which could be interpreted as “benign” inactivity in comparison to more severe immobility such as cast, bedrest, and spaceflight, is actually far more common in today's society and recapitulates the muscle deconditioning that is evident from these more extreme models of muscle disuse. This is an important health message given the recent (as of publication) shelter-at-home practices of ongoing pandemics (1). However, some potential nutritional strategies to minimize the loss of muscle mass and function could include greater protein intake (as suggested by Oikawa et al.), increased polyunstaturated fatty acids (PUFA's, as suggested by McGlory et al.), and/or creatine supplementation (as highlighted by Candow et al.), all of which may have greater efficacy in populations already at risk for low muscle mass and/or function such as the elderly and/or pre/post-operative patients. This discussion was also extended by Beaudry and Devries who highlighted the potential benefit of dietary protein (and potentially that which is dairy-based) and exercise (especially resistance exercise) in countering the metabolic dysregulation and low muscle quality common to clinical populations such as pre-diabetic (PD) and Type II diabetic (T2D) individuals. Incidentally, the original research of Sambashivaiah et al. reported lower muscle strength, but not mass, in PD and T2D Asian Indians compared to healthy controls, suggesting additional research into the habitual activity and dietary practices of pre- and clinical populations is warranted. Finally, obesity was discussed as a potential direct modulator of the anabolic resistance of skeletal muscle to both exercise and dietary protein by Beals et al., especially in conjunction with inactivity. Thus, these summative reviews represent important information for academics, knowledge translators, and knowledge end-users (e.g., clinicians and therapists) when identifying synergistic dietary and activity factors to maintain muscle mass and quality in vulnerable populations.

Dietary protein represents a primary nutrient for the remodeling of muscle tissue given its ability to independently stimulate muscle protein synthesis (2). However, Gwin et al. also demonstrated in healthy young adults entering military service that higher habitual protein intakes are associated with greater overall dietary quality and micronutrient ingestion, which generally supports previous recommendations that position nutrient dense, protein-rich whole foods as critical to maintain muscle health (3). Aside from total protein intake, Smeuninx et al. provided further evidence that individuals both young and old in the United Kingdom consume their daily protein in a skewed manner, highlighting the potential that redistributing protein from the larger evening meals to the morning may optimize muscle protein remodeling, providing a more efficient means to consume the daily protein intake. Snijders et al. also provided a comprehensive summary of the ability of pre-sleep protein ingestion to enhance nocturnal rates of muscle protein synthesis as a means to promote tissue remodeling and growth. Interestingly, the authors retrospectively assessed research from the van Loon laboratory at Maastricht University and demonstrated that greater protein intakes than that which maximize muscle protein synthesis in daytime meals (i.e., ~0.25 g/kg) (4) can dose-dependently (at least up to ~0.6 g/kg) support higher nocturnal muscle protein synthetic rates. This could suggest that a greater provision of amino acid substrates during an otherwise overnight fasted period are required to maximize muscle protein synthesis over an ~8 h sleeping period as compared to a daytime 4–6 h postprandial period. This apparently greater ability to assimilate dietary protein uptake into skeletal muscle during the overnight period could also explain in part the lack of difference in mixed muscle protein synthesis from ~0200 to 0800 h between 25 g of milk protein or a protein-free placebo consumed prior to bed (~2100 h) despite a positive ~10-h whole body net protein balance, as reported in this special issue by Karagounis et al.. Thus, daily protein redistribution independent of additional supplemental intake may represent a feasible means to optimize muscle mass and quality, especially if it arises from nutrient-dense sources.

With the deleterious effects of sarcopenia (loss of muscle mass and function) emerging as a significant health burden with the aging of much of the world's population (5), older adults represent a prime target for the development of strategies to maintain muscle health. At the forefront of nutritional strategies, protein intakes greater than the current recommended dietary allowance (RDA; 0.8 g/kg/d) are being advocated by many as a means to battle sarcopenia (6, 7). In this issue, Durainayagam et al. demonstrated that consuming twice the RDA for 10-week alters the metabolome in a manner that could be consistent with supporting increased tissue anabolism. With a growing interest in identifying responder phenotypes for personalized therapies, these results, if leveraged in larger cohorts, could serve as a springboard into additional trials that could advance this scientific and therapeutic aim. Further research from the Cameron-Smith lab as published by D'Souza et al. also demonstrated a potential role for micro-RNA (miR) species (i.e., miR-208a and−499a) in the regulation of the mechanistic target of rapamycin complex 1 (mTORC1) pathway after resistance exercise and protein ingestion, which may ultimate translate into differences in rates of muscle protein synthesis. As muscle protein synthesis may function to both resynthesize any old/damaged proteins broken down during the process of protein turnover as well as build new muscle proteins, optimizing this process in the elderly through exercise and/or nutritional approaches is of paramount importance for older adults. Original research by the Phillips laboratory, as presented by Bell et al. ostensibly supports this contention as integrated (i.e., “free-living”) rates of myofibrillar protein synthesis in overweight older adults over 24 h of post-exercise recovery were both enhanced with a multi-ingredient, protein-based supplement (i.e., whey, creatine, vitamin D, n-3 PUFA) and correlated with training-induced gains in lean body mass over 12-weeks of combined resistance and high-intensity interval training. Therefore, the present special issue provides important contributions to research and clinical endeavors that aim to maintain muscle mass and function with age.

The growth of new muscle and improvements in functionality (e.g., increased strength) are prime goals of many active individuals and especially athletes. The review by Slater et al. provides an excellent overview of the energy requirements for muscle hypertrophy as they discuss the variety of factors that must be considered when identifying the “sweet spot”, or minimum requirement, that both maximizes the growth of lean tissue with little to no concomitant fat mass growth. It is clear that ascribing to a “see-food” diet (i.e., unrestrained excess energy consumption) with resistance training will support muscle growth given the increased energy required for muscle contraction (i.e., training) and exercise-induced increases in muscle protein turnover (i.e., synthesis and breakdown). However, the authors highlight that current evidence suggests an additional ~1,500–2,000 kJ of additional energy may be a reasonable daily target to support muscle growth, although individual responses to this target may ultimately lead individuals to consider an *n* = 1 approach to nutrition (i.e., tinker with what “works” for them). Conversely, the maintenance of muscle mass and function is of importance for individuals aiming to optimize body composition (i.e., maximal fat loss) during energy restriction. Given the potential for n-3 PUFA's to increase muscle anabolism in some clinical populations (McGlory et al.), Philpott et al. explored the use of n-3 PUFA-enriched fish oil to help retain lean body mass and muscle strength during a short duration (i.e., 2 weeks) weight loss program in resistance trained males. They demonstrated that some measures of muscle strength (i.e., 1-repetition maximum knee extension) increased with fish oil with no concomitant retention of whole body fat-free mass during energy restriction. This research highlights the potential for n-3 PUFAs to be an adjuvant therapy for athletes aiming to maintain muscle function during targeted weight loss, possibly via enhanced neuromuscular function.

In addition to adequate energy, it is important to also consume adequate dietary protein to provide the amino acid substrates to support muscle protein remodeling and net protein synthesis during the post-exercise recovery period. A review in this issue (Moore) provides evidence that ~0.3 g of protein/kg body mass represents a dose that maximizes myofibrillar protein synthesis yet would minimize excess amino acid oxidative losses. Importantly, there is no evidence this target is influenced by sex or total active muscle mass (Moore), which increases the ease of translation across a range of body masses as compared to previous studies that provided absolute protein doses (8, 9). However, special consideration may need to be made for athletes engaging in very high volume (i.e., up to 32 sets/muscle group per training session) resistance training as gains in LBM over 6-weeks in trained males were enhanced by graded (i.e., from 25 to 150 g/day) whey protein ingestion (Haun et al.). This potentially highlights the need for additional research in highly active individuals who are not the typical untrained or recreationally active populations common to most basic/foundational research in this area. Original research from Edman et al. demonstrated that activation of mTORC1 (i.e., altered phosphorylation of S6K1 and eEF2) after exercise with essential amino acids is independent of muscle fiber type, suggesting that dietary amino acids are similarly anabolic in both type I and II fibers. Bridge et al. also demonstrate that Greek yogurt (providing ~20 g of protein) supports greater gains in lean body mass and some indices of strength over 12 weeks of training, providing further support for nutrient-dense whole foods as vital components of anabolic diets (3, 10). Thus, the articles in this special issue provide valuable information on the impact of dietary protein amount and type for active individuals aiming to enhance muscle anabolism, lean mass, and muscle strength.

Research advances in muscle biology may require the use of preclinical models, which can provide the foundational basis for the subsequent translation into human clinical trials. Caldow et al. demonstrated that the non-essential amino acid glycine can protect against inflammation-induced atrophy in C2C12 cells via an mTORC1-dependent mechanism. This research ultimately supports the importance of adequate intracellular glycine to offset catabolic muscle wasting conditions (e.g., cancer/inflammation). In addition to *in vitro* models, development of physiologically relevant *in vivo* models of resistance exercise could advance the study of contraction and nutrient interactions in mammalian skeletal muscle. To this end, D'Hulst et al. demonstrated that adding resistance to voluntary wheel running may be an ecologically valid model to study exercise-responses at the muscle level as compared to the robust (but perhaps less physiological) synergist ablation mouse models.

Skeletal muscle is exquisitely sensitive to the nutrients we eat and thus identifying dietary strategies that can enhance the growth or maintenance of this tissue are vital for individuals of all walks of life. While the quantity and quality of dietary protein and amino acids represent important factors regulating the synthesis of muscle proteins, research has also begun to investigate the impact of nutritive bioactives and non-protein factors that may independently regulate and/or augment normal postprandial muscle protein turnover. Furthermore, the mechanisms by which nutrition may propagate the stimulus for muscle remodeling and how it may control the transcription/translation of select genes is expanding at a rapid pace. Ultimately, identifying the dietary factors related to amount, type, and timing of nutrient ingestion that may promote muscle mass retention or gain are important components to “getting the most out of exercise” and supporting active living. With the contributions from world leaders in the field of nutrition, physical activity, and skeletal muscle biology, the current special issue represents a foundational repository of our current and emerging understanding of the role nutrition, in all its forms, plays in maintaining muscle health, quality, and performance across the lifespan.

## Author Contributions

All authors listed have made a substantial, direct and intellectual contribution to the work, and approved it for publication.

## Conflict of Interest

The authors declare that the research was conducted in the absence of any commercial or financial relationships that could be construed as a potential conflict of interest.
